# Supplementary material for: American crows that excel at tool use activate neural circuits distinct from less talented individuals
Source: Nat Commun. 2023 Oct 20;14:6539. doi: 10.1038/s41467-023-42203-8 (PMC10589215; doi:10.1038/s41467-023-42203-8)
Supplement: Supplementary file 1 — Supplementary Information [file 41467_2023_42203_MOESM1_ESM.pdf]

Supplementary Information

SUPPLEMENTARY METHODS

Behavior immediately before Imaging

To better gauge their level of attention towards the stimulus stage and to control for factors that may influence FDG uptake within the brain[1,2], we used a GoPro Hero 4 camera to record (30 fps) the gaze time, blink rate, and amount of movement of each crow during the stimulus phase of the imaging process. We were unable to obtain gaze and blink rate data from two birds' pre-training scan (n=1 from low proficiency and n=1 from medium proficiency groups) and a third bird's post-training scan (high proficiency) due to their eyes remaining out of the camera's field of view throughout the stimulus phase.

Avian brains are highly lateralized[3], so we measured the gaze time from each eye to verify that any observed differences in hemispherical activity were not due to the bird preferentially using one eye to view the stimulus over the other[4]. We tracked each eye's gaze time independently from the other eye e.g., we added gaze time to each eye if the bird binocularly gazed directly into the stage. We also used gaze time to measure a crow's level of interest in the stimulus being presented, and thus only recorded gaze when the stimulus stage's interior was revealed and visible to the crow (see *Imaging the crows*).

We measured blink rate to verify that the crows were not threatened by any of the presented stimuli or prior experience in the scanning apparatus, as previous studies have established a relationship between blink rate and the crow's perceived sense of danger; specifically, blink rate is negatively correlated with activity in fear-associated brain regions, and crows decrease blink rate when faced with a threatening stimulus compared to while foraging[1,2]. Although the image resolution was sufficient to see the white flash of the crow's nictating membrane, the birds sometimes turned their heads such that their eyes were no longer visible, so we calculated an observed blink rate by dividing the number of observed blinks by the amount of time the eye was visible. The cage interior became too dark to observe blinks when the panels to the stimulus stage were closed, so we only calculated blink rate when the stimulus stage was visible during the seven reveals of the stimulus phase.

We measured the crows' movement because physiological activity can confound the amount and location of FDG uptake within the brain[5]. We quantified the following actions as 1 unit of movement: crow moved 5-50 cm laterally along the perch (did not count if it moved <5cm, counted as 2 units if moved >50cm), crow rotated its body 180° to face the opposite direction, and crow hopping from the perch to the cage floor (or vice-versa). Because we measured movement to account for possible confounds to FDG uptake activity, we counted movement throughout the entire 10 minutes of the stimulus phase, including when the stimulus stage interior was hidden from the crow's view.

Supplementary Table 1. *Task proficiency and individual factors for all the crows used in this study.*

Bird ID	Mile-stone Level	Profi-ciency Level	Age	Sex	Level of Nervous (move/min)	Culmen Length (mm)	Abs. brain volume (cm^3)	Rel. brain volume (cm^3)	Brain volume % reduction	Capture Weight (g)	Release Weight (g)	Body Condition (g)
L-Or 2017	5	Med	Subadult	Female	14	47	6.8030	-0.443	5.50%	390	410	26.48
L-Re 2017	5	Med	Subadult	Male	23	52	8.2856	0.238	5.15%	435	505	27.339
L-Ye 2017	5	Med	Subadult	Male	42	51	7.3694	-0.518	5.58%	360	401	-38.833
R-Bl 2017	4	Low	Adult	Male	48	50	8.1852	0.458	7.88%	390	425	-0.005
R-Gr 2017	7	High	Adult	Female	36	45	6.7325	-0.193	7.81%	330	360	-15.864
R-Re 2017	1	Low	Adult	Male	38	51	7.5063	-0.381	9.01%	395	385	-3.833
R-Sk 2017	5	Med	Subadult	Male	37.5	52	7.8693	-0.179	4.51%	415	440	7.339
R-Wh 2017	5	Med	Subadult	Female	39	45	7.2686	0.343	6.39%	345	360	-0.864
L-Bl 2018	4	Low	Adult	Male	29.5	52	8.8306	0.783	2.46%	400	430	-7.661
L-Br 2018	5	Med	Subadult	Male	51.5	49	8.6737	1.107	9.15%	370	455	-11.177
L-Gr 2018	5	Med	Subadult	Male	38.5	50	7.7213	-0.006	3.58%	385	435	-5.005
L-Or 2018	7	High	Adult	Female	20.5	45	6.6071	-0.319	1.47%	355	385	9.136
L-Re 2018	7	High	Adult	Male	19.5	51	7.5450	-0.343	1.81%	425	460	26.167
L-Sk 2018	5	Med	Adult	Male	20.5	52	7.4936	-0.554	5.48%	400	420	-7.661
L-Wh 2018	4	Low	Subadult	Female	27.5	46	6.7218	-0.364	0.18%	335	410	-19.692
L-Ye 2018	7	High	Adult	Female	26	45	7.2969	0.371	7.27%	360	395	14.136

Supplementary Table 2. *Correlation matrix of the variables we obtained from the crows. We calculated Pearson's correlation coefficient when comparing two continuous variables and reported t-score and P-values for all comparisons for all variables. Sex and age are both categorical variables, so we did not include that comparison here (sex was exactly balanced across both age categories). Significant correlations are* ***bolded.***

	Relative brain volume	Absolute brain volume	Body condition	Culmen length	Nervous
Absolute brain volume	**r = 0.73**<br>**t_{14} = 3.98**<br>**P = 0.001**				
Body condition	r = 0.03<br>t_{14} = 0.12<br>P = 0.91	r = 0.02<br>t_{14} = 0.09<br>P = 0.93			
Culmen length	r = -3.77 e-10<br>t_{14} = -1.41 e-09<br>P = 1	**r = 0.68**<br>**t_{14} = 3.51**<br>**P = 0.003**	r = 9.35 e-12<br>t_{14} = 3.50 e-11<br>P = 1		
Nervous	r = 0.49<br>t_{14} = 2.08<br>P = 0.06	r = 0.43<br>t_{14} = 1.78<br>P = 0.10	**r = -0.58**<br>**t_{14} = -2.69**<br>**P = 0.017**	r = 0.11<br>t_{14} = 0.41<br>P = 0.69	
Sex	t_{14} = 0.62<br>P = 0.55	**t_{14} = 4.45**<br>**P < 0.001**	t_{14} = -0.37<br>P = 0.72	**t_{14} = 10.85**<br>**P < 0.001**	t_{14} = 1.39<br>P = 0.19
Age	t_{14} = 0.17<br>P = 0.87	t_{14} = 0.18<br>P = 0.86	t_{14} = -0.39<br>P = 0.70	t_{14} = 0.08<br>P = 0.94	t_{14} = 0.79<br>P = 0.44

Supplementary Table 3. *Crow proficiency levels and the associated task milestones they had to overcome as they learned to solve the task. Because all (but one) of the crows were ultimately distributed among three milestone levels (see Supplementary Table 1), we merged the seven milestone levels into three general proficiencies: crows that never dropped any training stones (levels 1-4) were low proficiency, crows that occasionally dropped training stones while they were balanced on the tube lip but never progressed further (level 5) were medium proficiency, and crows that fully mastered the task (level 7) were high proficiency. We classified any crows which reached high proficiency as having fully solved the task.*

Proficiency Level	Milestone Level	Criteria	Significance
None	0	Crows have not yet entered the training arena.	The starting point for naïve crows
Low	1	Exited the training cage, entered the white arena, ate food from the ground. Terminal proficiency for n=1	Crow overcame its neophobia of the training arena
	2	Ate from Aesop tube w/ water level 0 mm from the top (tube filled)	Crow overcame its neophobia of the Aesop tube
	3	Ate from Aesop tube w/ water level 50 mm from top	Crow overcame its reluctance to insert its head entirely inside the Aesop tube
	4	Maximum reachable distance established. Terminal proficiency for n=3	Maximum possible progression without dropping any stones into the tube
Med	5	Ate from the tube after dropping training stones balanced on the tube lip inside. Terminal proficiency for n=8	Crow has dropped a stone into the tube, but likely did so by accident
	6	Ate from the tube after lifting and dropping hanging training stones inside	Crow regularly observes that stones must be dropped inside before it can reach food
High	7	**Full mastery -** ate from the tube after lifting and dropping stones from the ground. Terminal proficiency for n=4	Crow associates the stones as necessary tools that must be dropped into the tube before it can retrieve food.

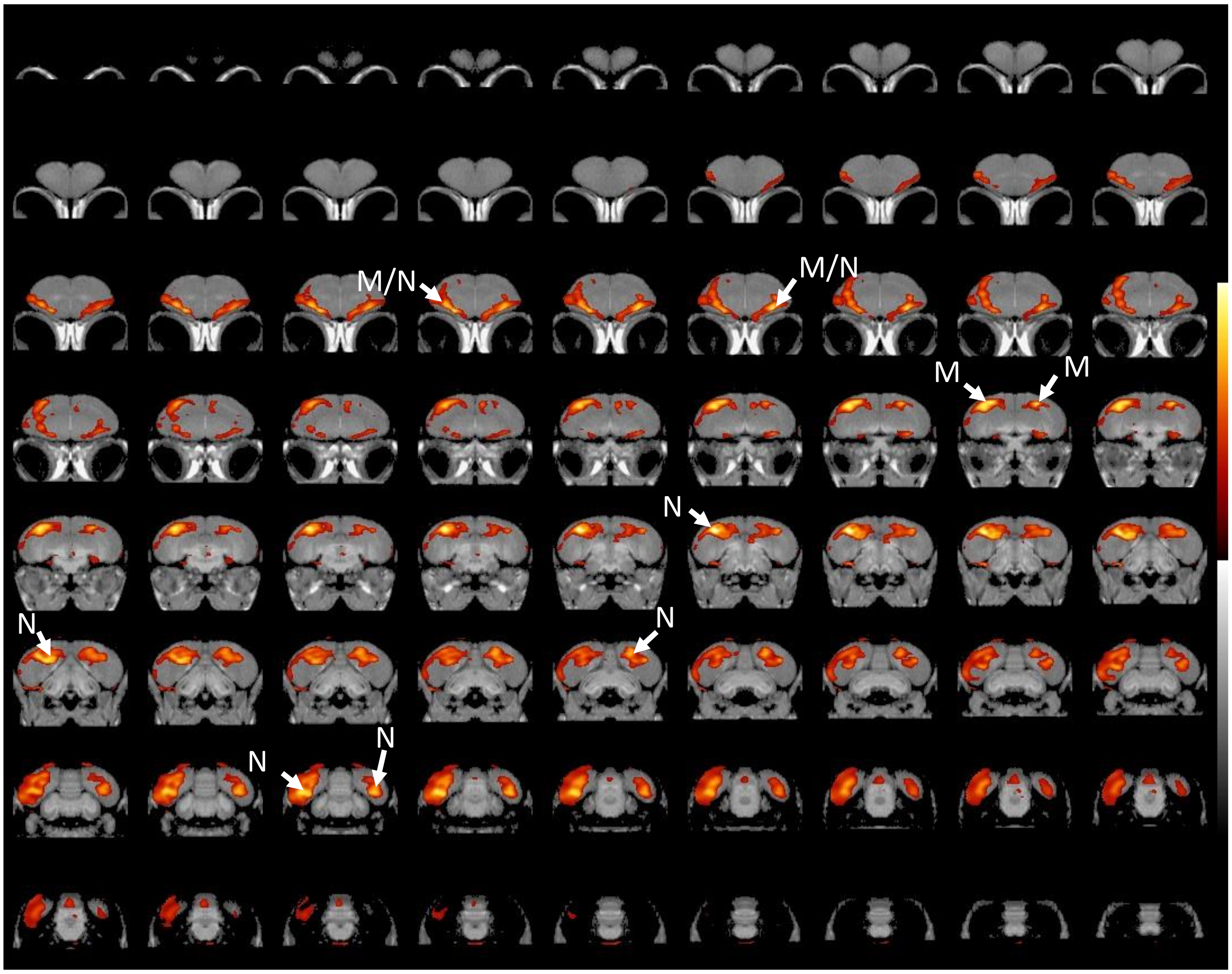

Supplementary Figure 1. *Coronal view of voxel-wise subtractions (converted to Z-score map) showing differential FDG uptake for all the crows' pre-training scan compared to their post-training scan (n=14 crows). M: mesopallium, N: nidopallium. The Z-score map is superimposed atop a composite (n=4 scans) structural MRI of the American crow brain. Note that the left hemisphere N activity at slice 49 (6th row, 5th column) did not exceed the Z-threshold for statistical significance. Source data are provided as a source data file.*

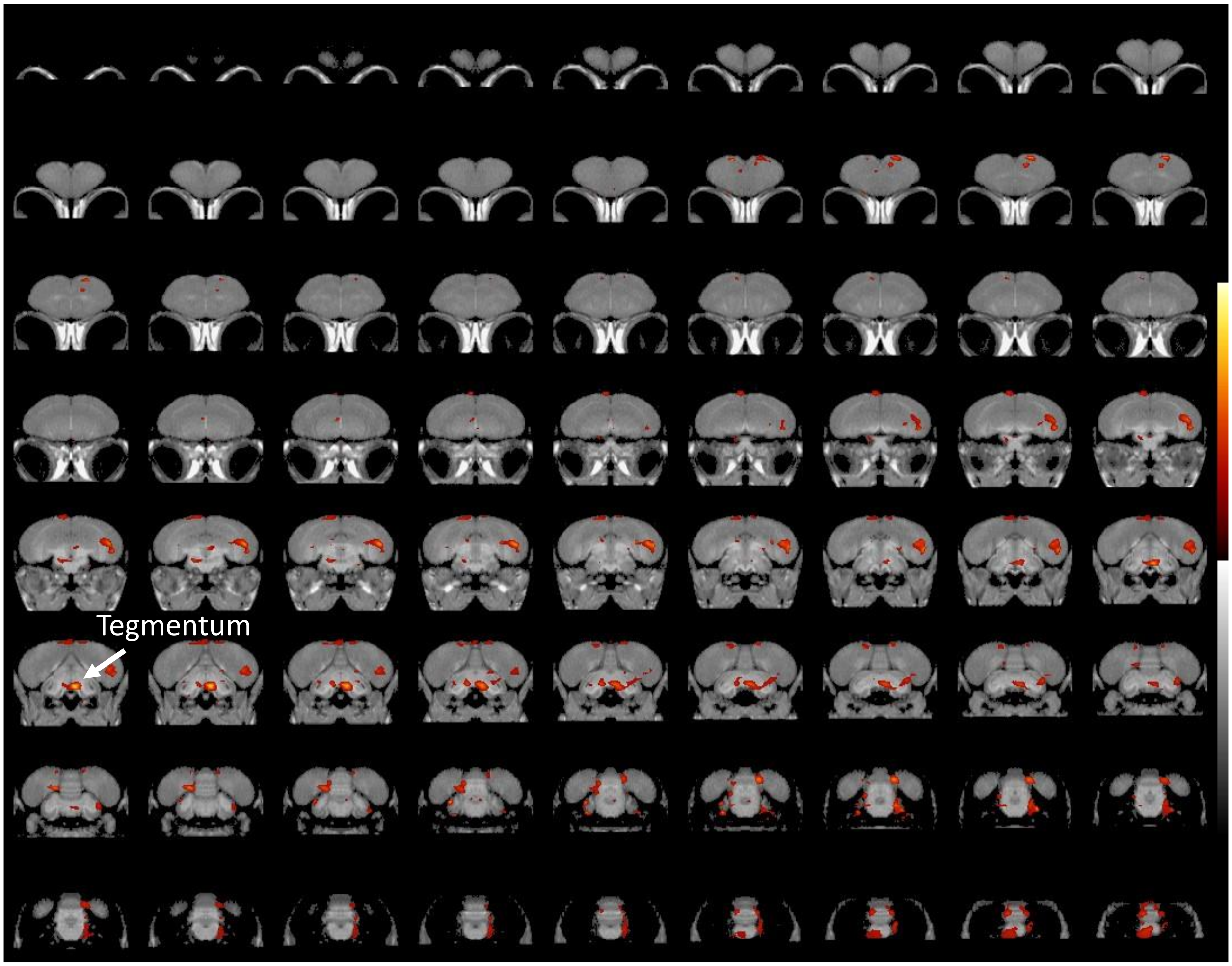

Supplementary Figure 2. *Coronal view of voxel-wise subtractions (converted to Z-score map) showing differential FDG uptake for the high task proficiency crows' post-training scan compared to their pre-training scan (n=4 crows). The Z-score map is superimposed atop a composite (n=4 scans) structural MRI of the American crow brain. Source data are provided as a source data file.*

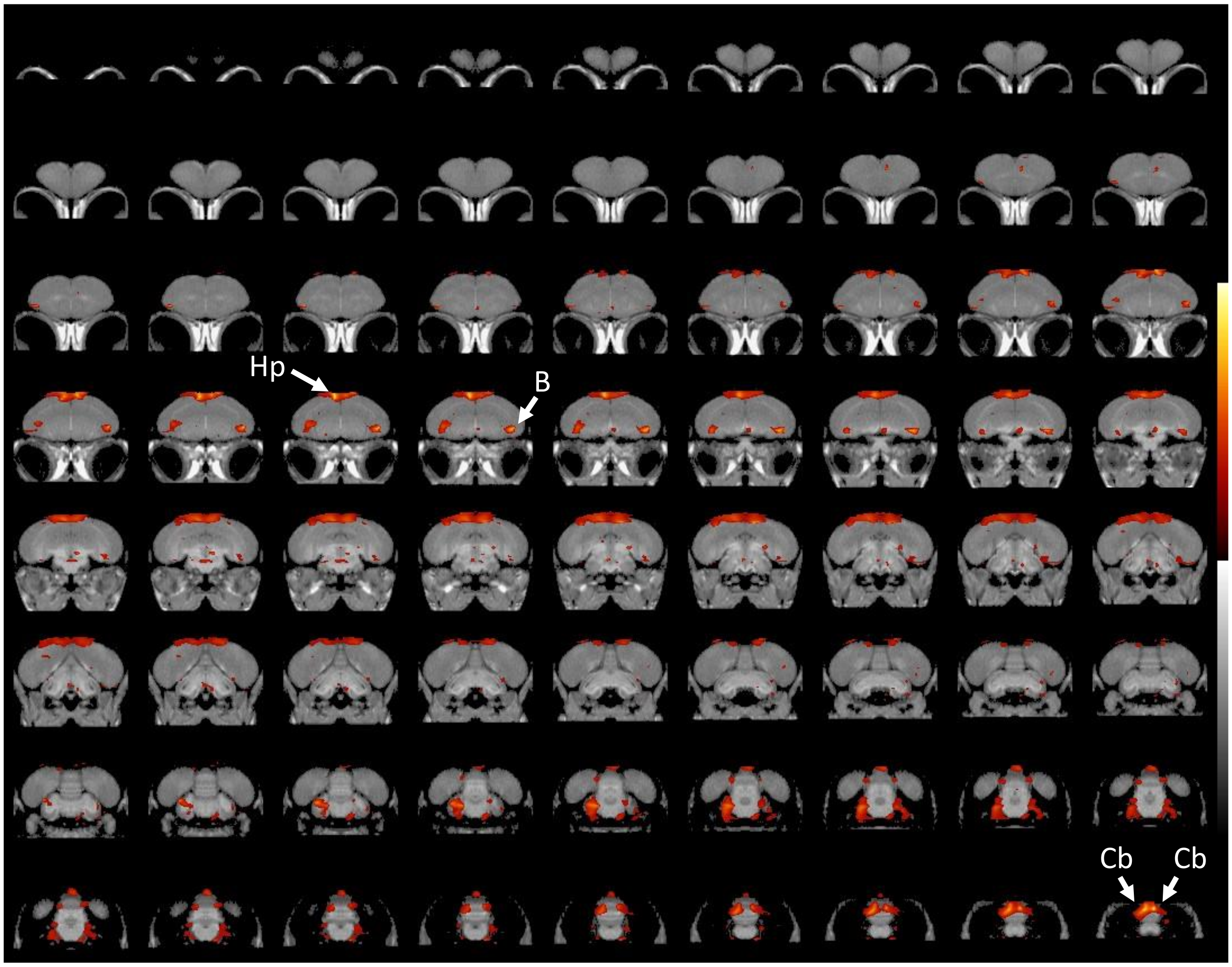

Supplementary Figure 3. *Coronal view of voxel-wise subtractions (converted to Z-score map) showing differential FDG uptake for the high proficiency crows' post-training scan compared to the same for low proficiency birds (n=7 crows). Hp: hippocampus, B: nucleus basorostralis, Cb: cerebellum. The Z-score map is superimposed atop a composite (n=4 scans) structural MRI of the American crow brain. Note that the nucleus basorostralis did not exceed the Z-threshold for statistical significance. Source data are provided as a source data file.*

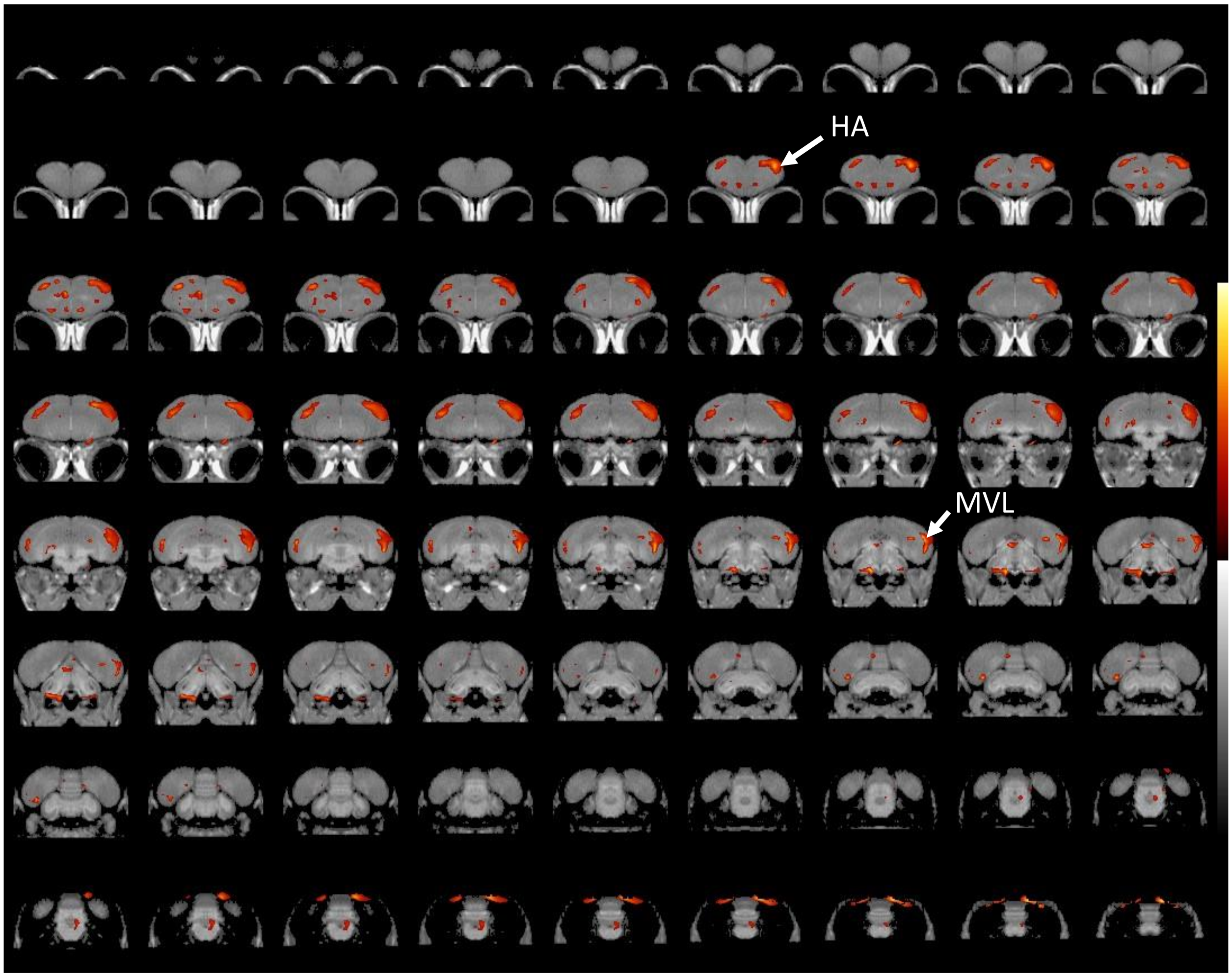

Supplementary Figure 4. *Coronal view of voxel-wise subtractions (converted to Z-score map) showing differential FDG uptake for the low proficiency crows' post-training scan compared to the same for high proficiency birds (n=7 crows). HA: hyperpallium apicale, MVL: mesopallium ventro-lateralis. The Z-score map is superimposed atop a composite (n=4 scans) structural MRI of the American crow brain. Note that neither region exceeded the Z-threshold for statistical significance. Source data are provided as a source data file.*

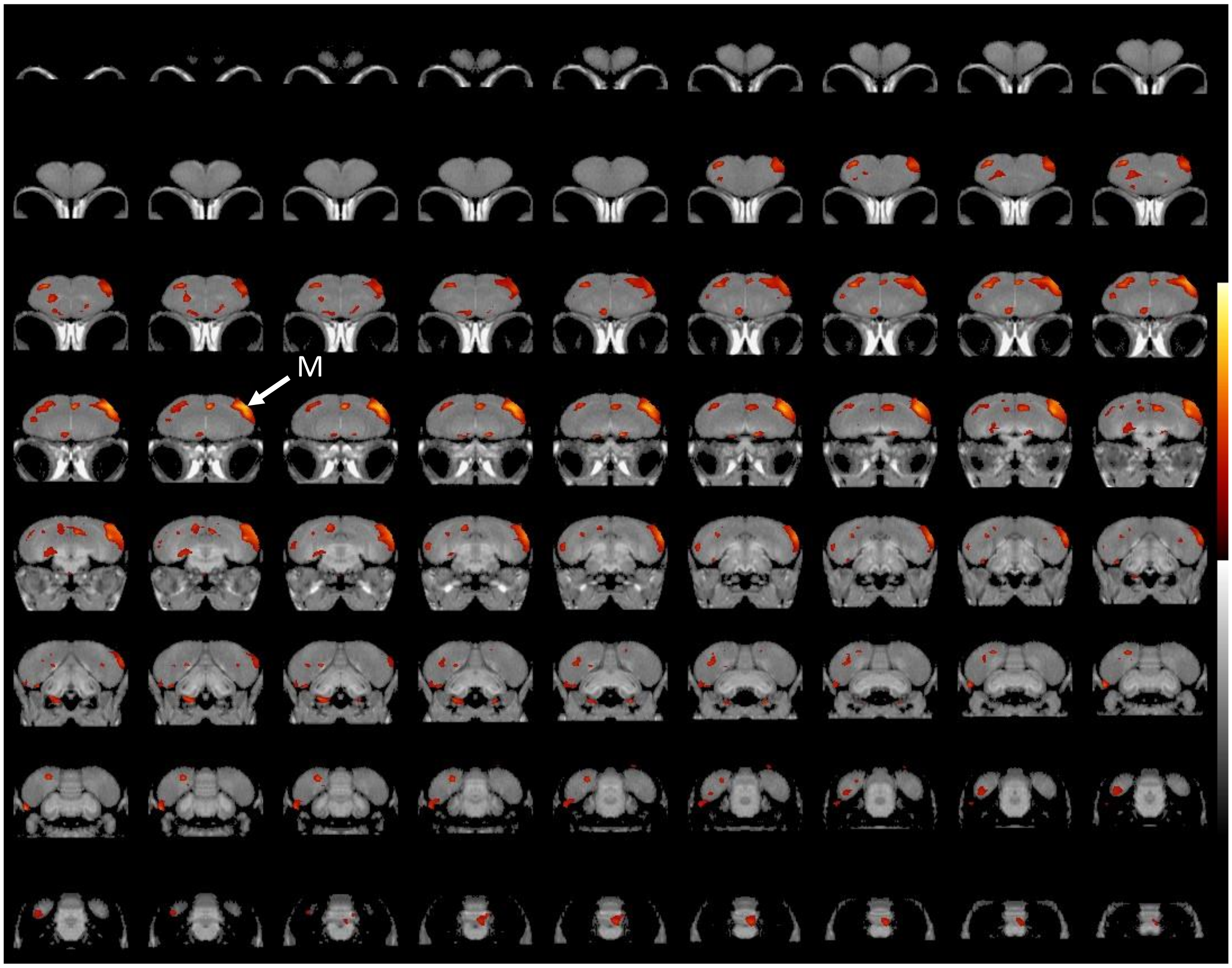

Supplementary Figure 5. *Coronal view of voxel-wise subtractions (converted to Z-score map) showing differential FDG uptake for the medium proficiency crows' post-training scan compared to the same for high proficiency birds (n=11 crows). M: mesopallium. The Z-score map is superimposed atop a composite (n=4 scans) structural MRI of the American crow brain. While notable, this region did not exceed the Z-threshold for statistical significance. Source data are provided as a source data file.*

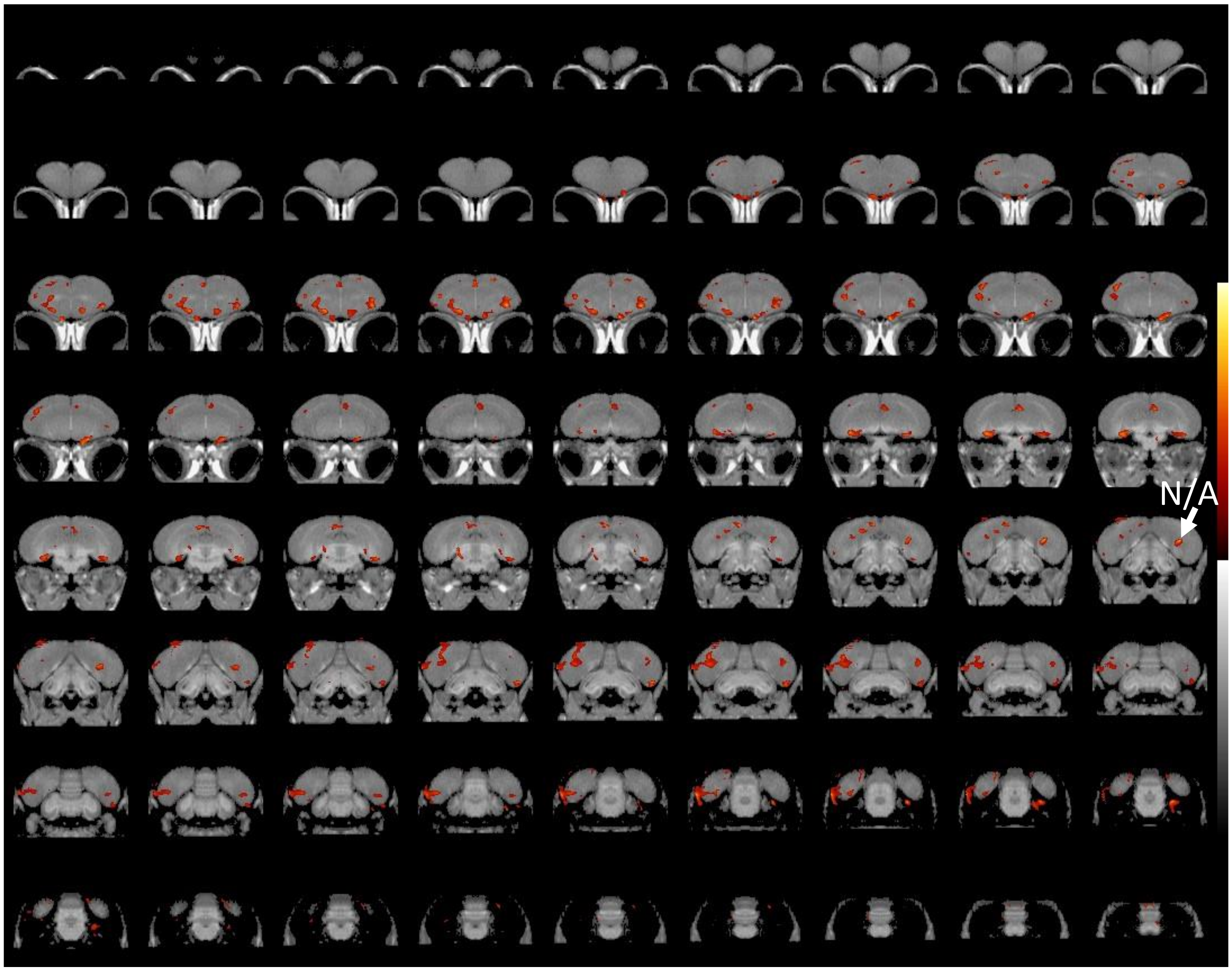

Supplementary Figure 6. *Coronal view of voxel-wise subtractions (converted to Z-score map) showing differential FDG uptake for the high proficiency crows' third scan (with full access to the Aesop's task apparatus) compared to their post-training scan (n=3 crows). N: nidopallium, A: arcopallium. The Z-score map is superimposed atop a composite (n=4 scans) structural MRI of the American crow brain.*

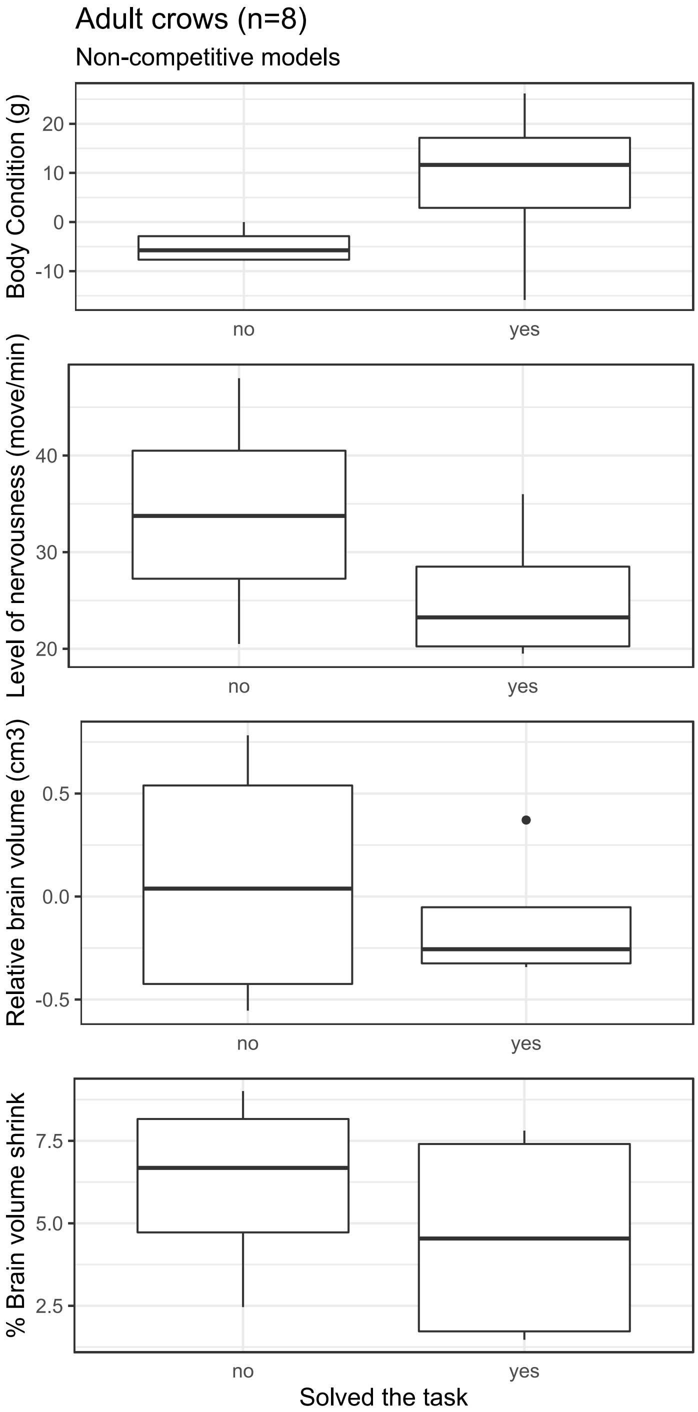

Supplementary Figure 7. *The remaining non-competitive variables attempting to predict which adult crows will solve the task: From top to bottom: body condition, level of nervousness, relative brain volume, and % reduction in brain volume between pre-training and post-training scan. Box-whisker plots display median (center line), upper/lower quartiles (box limits), and highest/lowest value within 150% of inter-quartile range (whiskers). Data beyond whiskers are displayed as outlier points. Source data are provided as a source data file.*

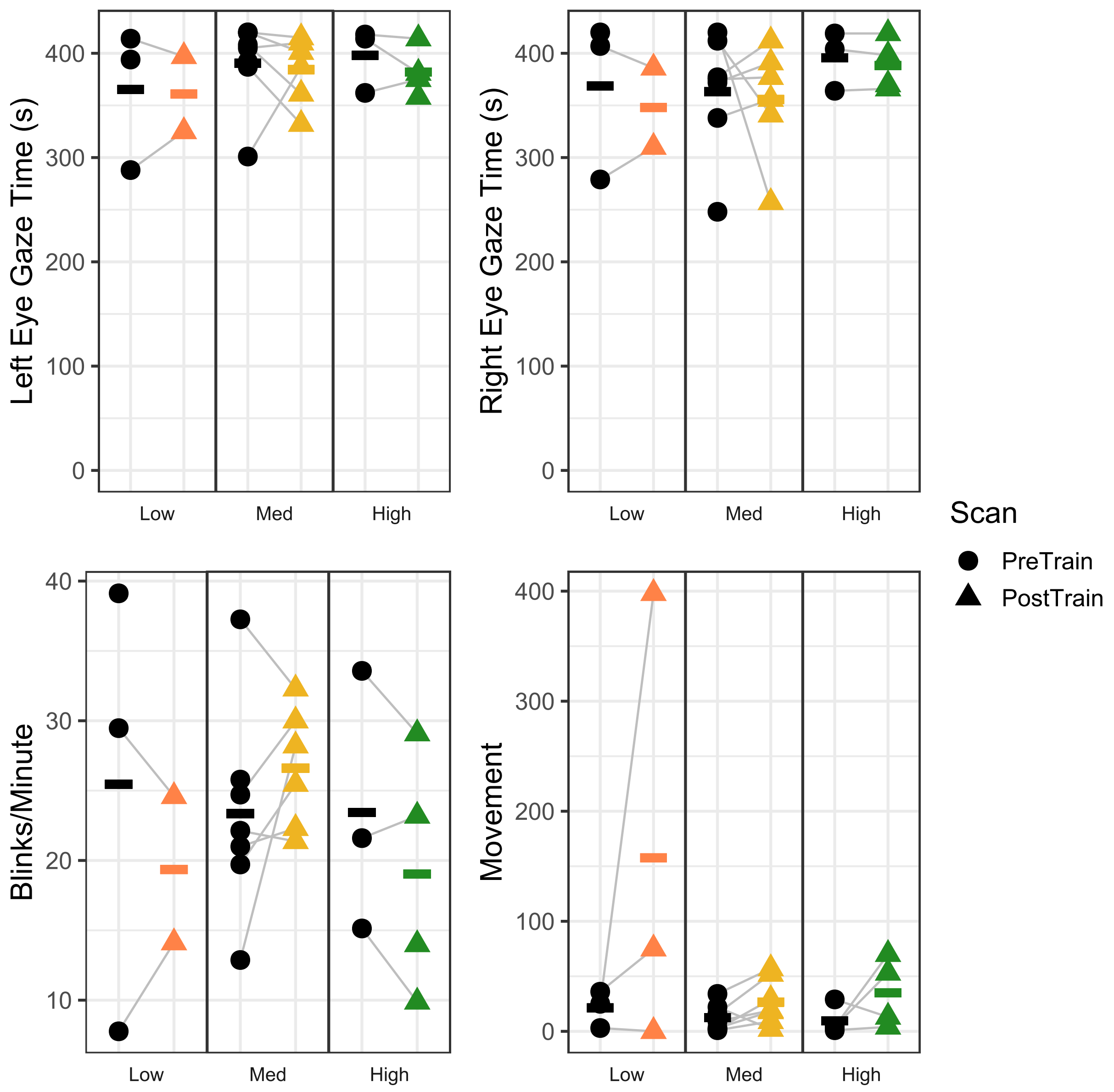

Supplementary Figure 8. *Changes in behavior between the first and second scan of each crow (n=14 crows) for blink rate (top left), total movement (top right), and gaze time (left eye: bottom left; right eye: bottom right). Graphs are subdivided by task proficiency. Horizontal lines indicate group means. Source data are provided as a source data file.*

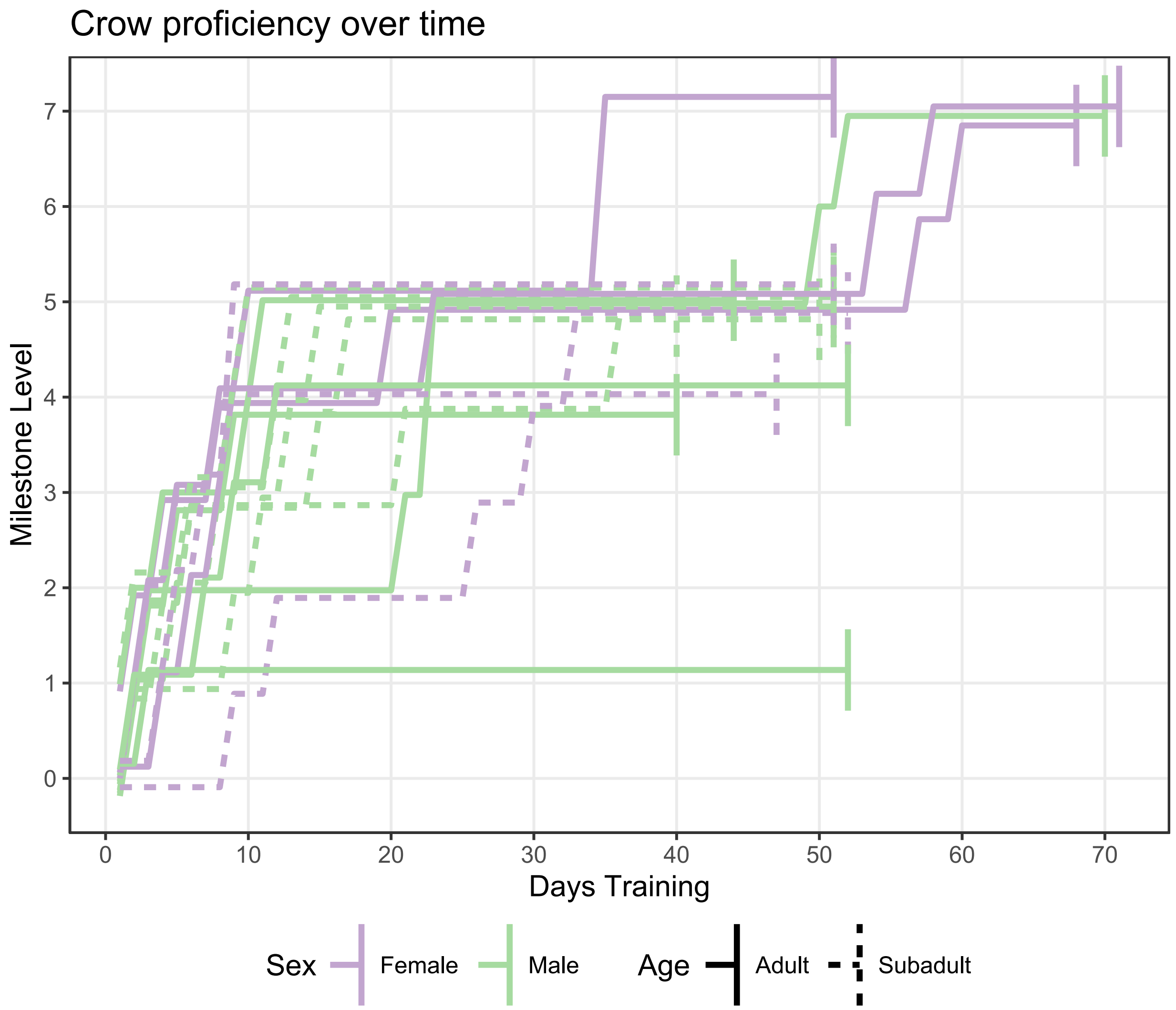

Supplementary Figure 9. *Learning curve tracking crow milestone level over time (see Supplementary Table 3 for details on milestone levels. Source data are provided as a source data file.*

SUPPLEMENTARY LITERATURE CITED

1. Cross, D. J. *et al.* Distinct neural circuits underlie assessment of a diversity of natural dangers by American crows. *Proc. R. Soc. Lond. B Biol. Sci.* **280**, 20131046 (2013).
2. Marzluff, J. M., Miyaoka, R., Minoshima, S. & Cross, D. J. Brain imaging reveals neuronal circuitry underlying the crow's perception of human faces. *Proc. Natl. Acad. Sci.* **109**, 15912–15917 (2012).
3. Rogers, L. J. & Anson, J. M. Lateralisation of function in the chicken fore-brain. *Pharmacol. Biochem. Behav.* **10**, 679–686 (1979).
4. Mench, J. A. & Andrew, R. J. Lateralization of a food search task in the domestic chick. *Behav. Neural Biol.* **46**, 107–114 (1986).
5. Bhargava, P., Rahman, S. & Wendt, J. Atlas of Confounding Factors in Head and Neck PET/CT Imaging. *Clin. Nucl. Med.* **36**, e20 (2011).
