# Supplementary material for: American crows that excel at tool use activate neural circuits distinct from less talented individuals
Source: Nat Commun. 2023 Oct 20;14:6539. doi: 10.1038/s41467-023-42203-8 (PMC10589215; doi:10.1038/s41467-023-42203-8)
Supplement: Supplementary file 2 — Reporting Summary [file 41467_2023_42203_MOESM2_ESM.pdf]

nature portfolio

Corresponding author(s): LomaJohn T Pendergraft

Last updated by author(s): Aug 31, 2023

Reporting Summary

Nature Portfolio wishes to improve the reproducibility of the work that we publish. This form provides structure for consistency and transparency in reporting. For further information on Nature Portfolio policies, see our Editorial Policies and the Editorial Policy Checklist.

Statistics

For all statistical analyses, confirm that the following items are present in the figure legend, table legend, main text, or Methods section.

n/a	Confirmed	
☐	☒	The exact sample size (n) for each experimental group/condition, given as a discrete number and unit of measurement
☐	☒	A statement on whether measurements were taken from distinct samples or whether the same sample was measured repeatedly
☐	☒	The statistical test(s) used AND whether they are one- or two-sided<br>*Only common tests should be described solely by name; describe more complex techniques in the Methods section.*
☐	☒	A description of all covariates tested
☐	☒	A description of any assumptions or corrections, such as tests of normality and adjustment for multiple comparisons
☐	☒	A full description of the statistical parameters including central tendency (e.g. means) or other basic estimates (e.g. regression coefficient) AND variation (e.g. standard deviation) or associated estimates of uncertainty (e.g. confidence intervals)
☐	☒	For null hypothesis testing, the test statistic (e.g. F, t, r) with confidence intervals, effect sizes, degrees of freedom and P value noted<br>*Give P values as exact values whenever suitable.*
☒	☐	For Bayesian analysis, information on the choice of priors and Markov chain Monte Carlo settings
☒	☐	For hierarchical and complex designs, identification of the appropriate level for tests and full reporting of outcomes
☐	☒	Estimates of effect sizes (e.g. Cohen's d, Pearson's r), indicating how they were calculated

Our web collection on statistics for biologists contains articles on many of the points above.

Software and code

Policy information about availability of computer code

Data collection	Raw PET/CT data was reconstructed to DICOM using a Siemens Inveon supplied 3D OSEM/MAP algorithm (reconstruction version 002.600). We stereotactically aligned all DICOM scans using custom algorithms originally designed for automated human brain analysis (NEUROSTAT). Note that NEUROSTAT does not use version numbers. These algorithms have been published and cited within our manuscript
Data analysis	We determined significant differences in brain activity using a custom voxel-wise subtraction and Z-statistic mapping algorithm originally designed for automated human brain analysis (NEUROSTAT). These algorithms have been published and cited within our manuscript. All other statistical analysis was conducted using R version 3.6.3

For manuscripts utilizing custom algorithms or software that are central to the research but not yet described in published literature, software must be made available to editors and reviewers. We strongly encourage code deposition in a community repository (e.g. GitHub). See the Nature Portfolio guidelines for submitting code & software for further information.

Data

Policy information about availability of data

All manuscripts must include a data availability statement. This statement should provide the following information, where applicable:

- Accession codes, unique identifiers, or web links for publicly available datasets
- A description of any restrictions on data availability
- For clinical datasets or third party data, please ensure that the statement adheres to our policy

The VOI coordinates, normalized uptake values, crow individual measures, crow training progress, and crow behavior during FDG uptake datasets generated in this

study have been deposited in the Dryad database and can be found here: https://datadryad.org/stash/share/OEUfMahmmZRnjzOicPtSc22AFaYbb26V7zdUMGLI1HY.

The jungle crow atlas mentioned in our manuscript can be found here: https://www.researchgate.net/profile/Ei-Ichi-Izawa/publication/230859490_A_stereotaxic_atlas_of_the_brain_of_the_jungle_crow_Corvus_macrorhynchos/links/02e7e5281b2b445293000000/A-stereotaxic-atlas-of-the-brain-of-the-jungle-crow-Corvus-macrorhynchos.pdf

The carrion crow atlas mentioned in our manuscript can be found here: https://onlinelibrary.wiley.com/doi/full/10.1002/cne.25392

Research involving human participants, their data, or biological material

Policy information about studies with human participants or human data. See also policy information about sex, gender (identity/presentation), and sexual orientation and race, ethnicity and racism.

Reporting on sex and gender	N/A
Reporting on race, ethnicity, or other socially relevant groupings	N/A
Population characteristics	N/A
Recruitment	N/A
Ethics oversight	N/A

Note that full information on the approval of the study protocol must also be provided in the manuscript.

Field-specific reporting

Please select the one below that is the best fit for your research. If you are not sure, read the appropriate sections before making your selection.

[x] Life sciences [ ] Behavioural & social sciences [ ] Ecological, evolutionary & environmental sciences

For a reference copy of the document with all sections, see nature.com/documents/nr-reporting-summary-flat.pdf

Life sciences study design

All studies must disclose on these points even when the disclosure is negative.

Sample size	We used 16 wild-caught American crows. We did not conduct any a priori analyses to determine sufficient sample size. Our sample size was limited by logistical constraints, such as the number of aviary cages we had at our disposal and the limited availability of the micro-PET/CT scanner
Data exclusions	We excluded two (n=2) PET scans from analysis because mechanical/software issues delayed the start of the scan beyond the 26 minute start time. Starting too early/late would affect the relative signal strength detected by the PET scanner due to the radioactive decay of FDG-18. We additionally excluded two (n=2) individuals from the stimulus phase blink count and gaze time because their position within the stimulus cage prevented the camera from seeing their eyes.
Replication	We did not make any efforts to replicate our results. Due to the limited funds available to us, the expense required to use the PET/CT scanner, and the time required to train new crows to solve the Aesop's fable apparatus, we focused our efforts on increasing our sample size instead of replicating our results.
Randomization	We captured our crows from the wild. Aside from releasing young birds at the moment of capture, our selection of crows to use in our study was random. We did not obtain any individual measures (sex, age, etc) from our crows until after we brought them into captivity and committed to using them in our study. Our sample size having sexes equally balanced by age (n=3 adult and 3 subadult females, n=5 adult and 5 subadult males) was due to chance.
Blinding	We were blind to our sample crows' sex, brain volume, and relative brain volume until after we obtained all scan data and released them. However, we were aware of age, size, body condition, and level of nervousness while we still had access to the birds. We were also aware of our crows' level of task proficiency as they progressed through daily training.

Reporting for specific materials, systems and methods

We require information from authors about some types of materials, experimental systems and methods used in many studies. Here, indicate whether each material, system or method listed is relevant to your study. If you are not sure if a list item applies to your research, read the appropriate section before selecting a response.

Materials & experimental systems

n/a	Involved in the study
[x]	[ ] Antibodies
[x]	[ ] Eukaryotic cell lines
[x]	[ ] Palaeontology and archaeology
[ ]	[x] Animals and other organisms
[x]	[ ] Clinical data
[x]	[ ] Dual use research of concern
[x]	[ ] Plants

Methods

n/a	Involved in the study
[x]	[ ] ChIP-seq
[x]	[ ] Flow cytometry
[x]	[ ] MRI-based neuroimaging

Animals and other research organisms

Policy information about studies involving animals; ARRIVE guidelines recommended for reporting animal research, and Sex and Gender in Research

Laboratory animals	No laboratory animals were used in this study
Wild animals	We captured 16 wild American crows from various locations in Seattle, Bothell, and Woodinville WA as they departed their communal roost by luring them with bread and then trapping them using a net launcher. We released all yearling individuals on site (identified by plumage and mouth coloration), and kept 8 subadults and 8 adults for use in our study. We transported crows using small animal kennels in an IACUC approved vehicle. We housed crows in a sheltered outdoor aviary at the University of Washington, Seattle. The crows were individually housed in adjacent cages (measuring 1.8 x 2.1 x 2.4 m) separated by wire mesh; crows could see and hear their neighbors but could not leave their cage. We provided crows with a rotating diet of assorted meats, eggs, grain, fruit, and dried dog kibble ad libitum for several hours each day. We released all crows back to the wild at the locations where we originally captured them at the conclusion of the study.
Reporting on sex	The sex of the crows was an important variable that we included in our analysis on the factors associated with task proficiency. Our sample of crows contained 10 males and 6 females. We sexed our birds using a QIAGEN® DNeasy® Blood & Tissue Kit to isolate genomic DNA from each blood sample, amplifying the target genes (CHD1-W and CHD1-Z) using polymerase chain reaction (PCR), and conducting agarose gel electrophoresis on the PCR product to reveal sex difference. Adult females are significantly more likely to master tool use compared to adult males.
Field-collected samples	We housed crows in a sheltered outdoor aviary at the University of Washington, Seattle. The crows were individually housed in adjacent cages (measuring 1.8 x 2.1 x 2.4 m) separated by wire mesh; crows could see and hear their neighbors but could not leave their cage. Crows received daily care, during which they were provided food, their cages were cleaned, and they were visually inspected for signs of injury, sickness, or distress. We provided crows with a rotating diet of assorted meats, eggs, grain, fruit, and dried dog kibble ad libitum for several hours each day. Because they were housed in a protected outdoor aviary, they were exposed to the same ambient conditions (temperature, photoperiod, etc.) as their wild conspecifics, albeit they received additional protection from precipitation (rain, snow, etc.). At the conclusion of the study, we transported the crows back to their original capture locations (using small animal carriers and IACUC approved vehicles) and released them back to the wild.
Ethics oversight	We captured, housed, and tested all crows (including PET/CT scans) in accordance with the Institutional Animal Care and Use Committee of the University of Washington (IACUC; protocol number 3077-01), Federal Collecting Permit MB761139-0, and State of Washington Scientific Collection Permit 14-010. All crows were released back into the wild after the study.

Note that full information on the approval of the study protocol must also be provided in the manuscript.
